# Evaluating effective population size and genetic diversity of a declining kit fox population using contemporary and historical specimens

**DOI:** 10.1002/ece3.4660

**Published:** 2018-11-08

**Authors:** Robert C. Lonsinger, Jennifer R. Adams, Lisette P. Waits

**Affiliations:** ^1^ Department of Natural Resource Management South Dakota State University Brookings South Dakota; ^2^ Department of Fish and Wildlife Sciences University of Idaho Moscow Idaho

**Keywords:** effective population size, gene flow, genetic diversity, kit fox, natural history collections, *Vulpes macrotis*

## Abstract

Loss of genetic diversity has serious conservation consequences (e.g., loss of adaptive potential, reduced population viability), but is difficult to evaluate without developing long‐term, multigenerational datasets. Alternatively, historical samples can provide insights into changes in genetic diversity and effective population size (*N*_e_). Kit foxes (*Vulpes macrotis*) are a species of conservation concern across much of their range. In western Utah, kit fox abundance has declined precipitously from historical levels, causing concern about population persistence. We analyzed genetic samples from museum specimens and contemporary scats to evaluate temporal changes in (a) genetic diversity and (b) *N*_e_ for kit foxes in western Utah, and (c) discuss our findings with respect to population risk and conservation. The *N*_e_ of kit foxes in western Utah has decreased substantially. When compared to established conservation thresholds for *N*_e_ (e.g., the 50/500 rule), observed levels suggest the population may be at risk of inbreeding depression and local extinction. In contrast, we found no significant decrease in genetic diversity associated with declining *N*_e_. We detected evidence of low levels of immigration into the population and suspect genetic diversity may have been maintained by this previously undescribed gene flow from adjacent populations. Low or intermittent immigration may serve to temper the potential short‐term negative consequences of low *N*_e_. We recommend that kit fox conservation efforts focus on evaluating and maintaining landscape connectivity. We demonstrate how historical specimens can provide a baseline of comparison for contemporary populations, highlighting the importance of natural history collections to conservation during a period of declining funding and support.

## 1 INTRODUCTION

Genetic diversity is a critical measure of biodiversity that impacts population viability (Frankham, 1996, 2005). Genetic diversity is influenced by population size, with smaller populations having an increased probability of inbreeding, genetic drift, and the potential fixation of deleterious alleles, which decreases genetic diversity and adaptive potential (Frankham, 1996; Hare et al., 2011; Palstra & Ruzzante, 2008). Effective population size (*N*_e_) is a theoretical measure of an idealized population size that would be expected to experience the same rate of genetic diversity loss (due to genetic drift) as the population under study (Wright, 1931). Effective population size is typically smaller than census population size, determines the rate at which genetic diversity declines in a population (Frankham, 2005; Hare et al., 2011), and is important for assessing the genetic health of a population and for predicting short‐term and long‐term risk (Palstra & Ruzzante, 2008). Small *N*_e_ and isolation (no or limited gene flow among populations) tend to accelerate stochastic loss of genetic diversity and can increase population risk and contribute to accelerated population loss (Fagan & Holmes, 2006; Gilpin & Soulé, 1986; Palstra & Ruzzante, 2008). Franklin (1980) suggested a minimum *N*_e_ ≥ 50 may be required to avoid short‐term inbreeding depression, but that an *N*_e_ ≥ 500 may be necessary to maintain long‐term adaptive potential. Concern over reductions in genetic diversity and *N*_e_ are further reinforced by their correlations with declines in population fitness (Reed & Frankham, 2003). Consequently, genetic diversity and *N*_e_ have important implications for species conservation and management (Frankham, 2005).

Populations of imperiled species are often small relative to their ancestral populations and likely suffer from decreased gene flow due to habitat fragmentation. Contemporary sampling can provide estimates of genetic diversity and *N*_e_ for species of concern, but conclusions may be misleading without a historical baseline. Despite the importance of interpreting genetic parameters with respect to a historical baseline for conservation, evaluating changes during population declines can be notoriously difficult due to the need for long‐term studies or well‐preserved historical samples. Natural history collections (NHCs) can therefore be a critical resource to conservation, providing a retrospective assessment of populations (Graham, Ferrier, Huettman, Moritz, & Peterson, 2004; Holmes et al., 2016; Lister et al., 2011; McLean et al., 2016). For example, low genetic diversity and small *N*_e_ exhibited by Yellowstone grizzly bears (*Ursus acrtos*) suggested the population may have suffered a bottleneck and were at imminent risk of reduced viability (Miller & Waits, 2003). Yet, evaluating historical samples revealed that genetic diversity was historically low and had been declining at a rate lower than previously suspected, suggesting the grizzly bear population was unlikely to be at imminent risk due to genetic factors (Miller & Waits, 2003).

Kit foxes (*Vulpes macrotis*) are a species of conservation concern across much of their historical range (Dempsey, Gese, Kluever, Lonsinger, & Waits, 2015; Eckrich et al., 2018; Lonsinger, Gese, Bailey, & Waits, 2017; Lonsinger, Lukacs, Gese, Knight, & Waits, 2018). Native to western North America, kit foxes are among the smallest canids and are adapted to arid desert habitats (Egoscue, 1962, Golightly & Ohmart, 1984; see McGrew (1979) for a historical distribution map). In the Great Basin Desert, kit foxes were abundant in the mid‐1900s (Egoscue, 1956, 1962 ). Since 1970, changes in landscape and community dynamics have altered the habitat for kit foxes. Anthropogenic water developments have increased water availability (Arjo, Gese, Bennett, & Kozlowski, 2007), annual grasslands (primarily invasive cheatgrass [*Bromus tectorum*]) have increased in distribution, and wildfires have increased in frequency (Sparks, West, & Allen, 1990). These landcover changes have influenced rodent (i.e., prey) communities (Smith, Gese, & Kluever, 2017). Furthermore, black‐tailed jackrabbits (*Lepus californicus*; an important prey) have declined, and coyotes (*Canis latrans*; a dominant intraguild predator) have increased (Arjo et al., 2007; Byerly, Lonsinger, Gese, Kozlowski, & Waits, 2018). Consequently, kit fox density has declined significantly from historical levels to one of the lowest densities reported across their range (Lonsinger et al., 2018).

Despite considerable effort to understand the ecology of kit foxes in the Great Basin Desert (e.g., Arjo, Bennett, & Kozlowski, 2003; Egoscue, 1956, 1962, 1975 ) and the impacts of changing landscape and community dynamics (e.g., Arjo et al., 2007; Byerly et al., 2018; Kluever & Gese, 2017; Kozlowski, Gese, & Arjo, 2012; Lonsinger et al., 2017), there has been a paucity of research into the genetic health of the kit fox population. Understanding the population genetic health of kit foxes is essential for developing effective conservation strategies. To this end, this study (a) investigated contemporary genetic diversity and *N*_e_ for kit foxes in western Utah and (b) compared these findings to estimates based on historical kit fox specimens collected before major landscape and community changes occurred (i.e., before 1970) to evaluate changes. Considering the precipitous decline in kit fox densities, we hypothesized that genetic diversity would be decreased in the contemporary population relative to the historical population. Similarly, we expected that contemporary *N*_e_ would be significantly lower than historical *N*_e_. Based on recent abundance estimates (Lonsinger et al., 2018) and the general relationship between census population size and *N*_e_, we hypothesized that contemporary estimates of *N*_e_ would be below the critical threshold of *N*_e_ = 50 suggested to avoid short‐term effects of inbreeding. We discuss our findings with respect to the management and conservation of kit foxes in the Great Basin and highlight the importance of NHCs to conservation.

## 2 MATERIALS AND METHODS

### 2.1 Study area

We focused on kit fox populations in western Utah within and around the U.S. Army's Dugway Proving Ground (Dugway). The region is characterized as Great Basin Desert with low‐lying basins separated by mountains. Habitat varies from playa, vegetated and unvegetated dunes, grasslands, and shrublands at lower elevations, to shrubland and open woodland at higher elevations (Arjo et al., 2007). The kit fox population in this region provided a unique opportunity to evaluate changes in genetic diversity and *N*_e_, due to the long history of kit fox research and associated collection of historical samples within the region (Egoscue, 1956, 1962, 1975).

### 2.2 Contemporary and historical genetic sampling

We collected contemporary kit fox samples through noninvasive genetic sampling intended to estimate patterns of occupancy (Lonsinger et al., 2017) and density (Lonsinger et al., 2018) of kit foxes. We conducted carnivore scat surveys along two‐track and gravel roadways over 2 years (2013–2014), including two winter (January–March) and two summer (July–August) seasons (Lonsinger et al., 2018). We collected fecal material from the side of each scat for genetic analysis. Sampling methods are detailed in Lonsinger et al. (2018).

We collected historical kit fox samples from specimens housed in the Natural History Museum of Utah from 1951–1969. When available, we collected samples from the maxilloturbinates (nasal bones), tentorium and internal occipital protuberance (cranial bones), and toepads of each kit fox specimen (Casas‐Marce, Revilla, & Godoy, 2010; Miller & Waits, 2003; Wisely, Maldonado, & Fleischer, 2004). As is common with the application of historical specimens, the museum preparation history of our specimens was unknown and likely varied by researcher and over time. We removed nasal and cranial bone samples with sterilized tweezers or forceps, and toepads with a sterile razor blade. All sampling procedures aimed to minimize damage to the specimens and were approved by the Natural History Museum of Utah. Samples were weighed, placed in coin envelopes, and stored with silica desiccant until DNA extraction.

### 2.3 Laboratory procedures

We restricted DNA extraction and polymerase‐chain reaction (PCR) setup to dedicated laboratories to minimize contamination risk. For historical samples, DNA extraction and PCR set up were conducted in a laboratory that had not previously been used to house or process vertebrate DNA (including noninvasive samples) and was spatially separated (i.e., different buildings) from areas in which DNA amplification was performed. Noninvasive contemporary samples were processed (i.e., DNA extraction and PCR set up) in a laboratory dedicated to low quality samples that was also spatially separated (i.e., different floors) from areas in which DNA amplification was performed. Protocols restricted movement of supplies, equipment, and people from the historical to noninvasive laboratories, and from the noninvasive to postamplification laboratories (Waits & Paetkau, 2005). For contemporary samples, DNA storage, extraction, amplification, and scoring methods are detailed in Lonsinger, Gese, and Waits (2015). We determined species identification of contemporary samples using a mitochondrial DNA fragment analysis test (De Barba et al., 2014). For historical samples, we extracted DNA from ~0.06 g of each sample. If <0.06 g was available, we extracted DNA from the entire sample. We used liquid nitrogen and a sterilized mortar and pestle to grind bone samples into a powder. We sliced toepads into the smallest pieces possible with a sterile razor. We extracted DNA from each historical sample using the “silica” method (Boom et al., 1990; Höss & Pääbo, 1993). We included a negative control with each extraction set (i.e., each batch of ~19 samples) to monitor for contamination.

We amplified kit fox samples with nine nuclear DNA (nDNA) microsatellite loci (Cullingham, Smeeton, & White, 2006; Francisco, Langston, Mellersh, Neal, & Ostrander, 1996; Fredholm & Wintero, 1995; Holmes et al., 1995; Ostrander, Mapa, Yee, & Rine, 1995; Ostrander, Sprague, & Rine, 1993) and two sex identification primers (Berry, Sarre, Farrington, & Aitken, 2007). Primers were combined into a single multiplex. For contemporary scat samples, the PCR conditions for the 7 μl (total volume) multiplex for each primer pair were 0.29 μM CXX103, 0.09 μM VVE‐M19, 0.06 μM FH2054, 0.04 μM CXX250, FH2001, FH2010, and CPH3, 0.03 μM FH2088 and CF‐hprt, and 0.01 μM CXX377 and VV‐sry, combined with 1× concentrated Qiagen Master Mix, 0.5× concentrated Q solution and 1 μl of DNA extract. For historical samples, PCR conditions for the 7 µl (total volume) multiplex were 0.29 µM CXX103, 0.09 µM VVE‐M19, 0.06 µM FH2054, 0.29 µM CXX250, 0.04 µM FH2001, FH2010, and CPH3, 0.03 µM FH2088, 0.04 µM CF‐hprt, 0.03 µM CXX377, and 0.06 µM VV‐sry, combined with 1× concentrated Qiagen Master Mix, 0.5× concentrated Q solution, and 1 µl of DNA extract.

For contemporary samples, the PCR thermal profile had an initial denaturation of 94°C for 15 min, 15 touchdown cycles at 94°C for 30 s (denaturation), 63°C for 90 s (annealing; decreasing by 0.5°C per cycle), and 72°C for 60 s (elongation), 20 cycles at 94°C for 30 s (denaturation), 55°C for 90 s (annealing), and 72°C for 60 s (elongation), and a final elongation at 60°C for 30 min. For historical samples, the PCR thermal profile had an initial denaturation of 95°C for 15 min, 15 touchdown cycles at 94°C for 30 s (denaturation), 63°C for 90 s (annealing; decreasing by 0.5°C per cycle) and 72°C for 60 s (elongation), 35 cycles at 94°C for 30 s (denaturation), 55°C for 90 s (annealing), and 72°C for 60 s (elongation), and a final elongation at 60°C for 30 min.

We conducted all PCR procedures for contemporary and historical samples on a Bio‐Rad Tetrad thermocycler (Bio‐Rad, Hercules, CA, USA) with negative and positive controls. We used a 3130×l DNA Analyzer (Applied Biosystems, Foster City, CA, USA) to obtain results and genemapper 3.7 (Applied Biosystems) to visualize and score allele sizes.

To minimize genotyping errors in contemporary samples, we dropped low‐quality samples that failed species identification (Kohn et al., 1999) and used a multi‐tubes approach for nDNA analyses (Taberlet et al., 1996). We initially amplified samples in duplicate, culling lower quality samples that amplified at <50% of loci (Paetkau, 2003). We then performed additional replicates for retained samples until consensus genotypes were achieved across loci or we reached eight replicates. We established consensus genotypes by comparing replicates with congenr (Lonsinger & Waits, 2015) and requiring alleles of heterozygous and homozygous alleles to be observed ≥2 and ≥3 times, respectively. To achieve a probability of identity for siblings (i.e., probability that two siblings have identical multilocus genotypes; P(ID)sibs; Waits et al., 2001) <0.01, consensus genotypes were required at ≥6 loci (excluding sex identification markers; Lonsinger et al., 2018). We dropped samples that failed to achieve a P(ID)sibs <0.01. Similarly, for historical samples, we employed a multitubes approach. We ensured that ≥3 replicates were performed per sample source (i.e., cranial bones, nasal bones, and/or toepads). We increased the number of replicates as necessary until we achieved consensus genotypes at a sufficient number of loci for each specimen (i.e., each individual kit fox, considering all available sample sources), or until we reached a maximum of six replicates per sample source for samples with successful amplification at approximately ≥50% of loci. We estimated genotyping error rates by comparing each replicate to its respective consensus genotype with congenr (Lonsinger & Waits, 2015). An allele observed in a replicate but not in the consensus genotype was recorded as a false allele, whereas an allele observed in the consensus genotype but not in a replicate with successful amplification was recorded as allelic dropout.

### 2.4 Genetic diversity and population genetic structure

We restricted genetic analyses of contemporary samples to kit foxes detected during winter, or that were known to have survived over winter (i.e., samples detected in each summer). This restricts analyses to those individuals that survived until at least their first breeding season and therefore had the opportunity to contribute to the breeding population. For historical samples, we restricted our analyses to samples with date and location (county) of collection data, and where the location aligned with (Tooele County, Utah), or was adjacent to (Juab County, Utah), our contemporary sampling area. Closely related individuals may bias the results of some genetic analyses (Anderson & Dunham, 2008). Thus, we evaluated pairwise relatedness (Queller & Goodnight, 1989) among individuals within historical and contemporary kit fox samples with genalex v6.5 (Peakall & Smouse, 2006) and removed one individual from each pair with a coefficient of relatedness ≥0.45 (Louis et al. 2014). Hale, Burg, and Steeves (2012) demonstrated that sampling 25 to 30 individuals from a population is sufficient to characterize population‐level genetic diversity when using microsatellites. Following the removal of closely related individuals, our sample sizes exceeded these sampling requirements (see Results).

We tested for departure from Hardy–Weinberg Equilibrium (HWE) and linkage equilibrium across loci for historical and contemporary populations with the probability test in genepop v4.2 (Raymond & Rousset, 1995) with Bonferroni's corrections (Rice, 1989). As our historical samples spanned 19 years, we split the historical samples into two (1951–1959 and 1961–1969) and three (1951–1955, 1958–1962, and 1964–1969) groups temporally, and evaluated differences in allele frequencies between groups with the *G* test in genepop (Raymond & Rousset, 1995). We did not detect significant changes in allele frequencies over the historical samples (see Results) and we therefore considered all historical samples as a single population characterizing the kit fox population prior to its decline in density (Lonsinger et al., 2018).

Although we expected the spatial extent of historical samples to represent a single population, historical locations were recorded at the county level and the exact locations were unknown. To test for population genetic structure within the historical and contemporary samples, we used the program structure v2.3. (Pritchard, Stephens, & Donnelly, 2000). structure employs Bayesian clustering techniques to infer the most likely number of genetic clusters (*K*) that best reflect HWE and linkage equilibrium. For each sample (historical and contemporary), we performed 10 independent runs of structure, identifying the most supported number of *K* from a range of *K = *1–4 clusters. We used the admixture model with correlated alleles. Each run included 50,000 burn‐in and 50,000 Markov Chain Monte‐Carlo iterations; runtime evaluations of summary statistic stability suggested that these run lengths were sufficient (Pritchard et al., 2000). We inferred the most probable *K* from each analysis based on the maximum mean log likelihood (L[*K*]; Pritchard et al., 2000).

We calculated genetic diversity measures, including observed (*H*_O_), Nei's unbiased expected heterozygosity (*H*_E_), and the inbreeding coefficient (*F*_IS_), independently for contemporary and historical populations with genalex (Peakall & Smouse, 2006). We calculated allelic richness (*A*_r_) with fstat v2.9.3.2 (Goudet, 1995). We tested for differences in *H*_E_ and *A*_r_ across loci between historical and contemporary populations with a paired two‐tailed *t*‐test in the R programming language (R Core & Team, 2018). We evaluated differences in allele frequencies between historical and contemporary populations with the *G* test in genepop (Raymond & Rousset, 1995).

### 2.5 Effective population size

We used both two‐sample (i.e., temporal) and single‐sample methods to estimate kit fox *N*_e_. Temporal methods estimate the variance *N*_e_ and generate a harmonic mean *N*_e_ over generations between sampling periods (Hare et al., 2011; e.g., in our case, over generations between our historical and contemporary periods). Single‐sample linkage disequilibrium methods estimate inbreeding *N*_e_ and provide point estimates for the *N*_e_ of the preceding generation (Hare et al., 2011). We used two formulations of the temporal method, *F*_k_ (Pollak, 1983) and *F*_c_ (Nei & Tajima, 1981), as well as the linkage disequilibrium single‐sample method (Waples, 2006; Waples & Do, 2008) as implemented in neestimator v2.1 (Do et al., 2014). Sample size is an important consideration when estimating *N*_e_ and small sample sizes can result in large biases (Waples & Yokota, 2007). Consequently, we used all individuals (including individuals identified as siblings) in our analyses of *N*_e_. This resulted in sample sizes (historical = 49; contemporary = 76) that have been shown to generally produce accurate and precise estimates (Waples & Yokota, 2007). Although the impact of close relatives on *N*_e_ estimates is not entirely understood, removing siblings can weaken the signal used to infer *N*_e_, and the linkage disequilibrium method showed little bias with siblings included (Waples & Anderson, 2017). Kit fox generation time was estimated to be ~3.4 years (Kelly, Allred, Possingham, & Williams, 1995). We estimated that there were 13 generations between historical and contemporary sampling and used this when estimating *N*_e_ with temporal methods. Kit foxes are primarily monogamous (Ralls, Cypher, & Spiegel, 2007), and we set the mating model accordingly for the linkage disequilibrium method. Simulations suggested that inclusion of rare alleles can bias estimates of *N*_e_ (Do et al., 2014). We considered two critical values (*P*_crit_ = 0.01 and 0.05) and filtered out rare alleles occurring at frequencies lower than these values. We generated 95% confidence intervals based on the jackknife‐across samples method (Jones, Ovenden, & Wang, 2016) with neestimator v2.1 (Do et al., 2014). The bias correction methods for handling missing data in neestimator v2.1 assumes that missing values are independent and random (Do et al., 2014; Peel, Waples, Macbeth, Do, & Ovenden, 2013). Over half of our missing data occurred at a single locus (CXX250), and our historical population was missing data for nearly 30% of individuals at this locus. We removed CXX250 and performed the *N*_e_ analyses based on the remaining eight loci. For resulting *N*_e_ estimates from each method, we calculated the harmonic mean across critical values.

### 2.6 Identification of Immigrants

Our results suggested that genetic diversity may have been maintained by immigration (see Results). We tested our historical and contemporary populations for immigrants by evaluating the likelihood of each individual's multilocus genotype within their respective population using the leave‐one‐out method implemented in geneclass2 v2.0 (Piry et al., 2004). We applied the frequencies‐based computational method (Paetkau, Calvert, Stirling, & Strobeck, 1995) and calculated the probability that at each individual is an immigrant with Monte‐Carlo resampling based on 10,000 simulated individuals (Paetkau, Slade, Burden, & Estoup, 2004).

## 3 RESULTS

### 3.1 Kit fox sampling and individual identification

We surveyed 570–870 km of transects during each season and collected 810 scats confirmed as kit fox with mitochondrial DNA. Of these, we identified 76 (M:F ratio = 1.7:1) contemporary individuals and achieved complete multilocus consensus genotypes for 91% of individuals; individuals without complete genotypes had consensus genotypes at an average of 7 (±0.40 *SE*) loci (range = 5–8). We collected 120 samples including 45 cranial bones, 47 nasal bones, and 28 toe pads from 56 historical specimens. Seven specimens failed to produce sufficient amplifications and were dropped. Of the 49 (M:F ratio = 1.9:1) historical individuals retained, 69% achieved complete multilocus consensus genotypes across loci; individuals without complete multilocus genotypes had consensus genotypes at an average of 7.8 (±0.14 *SE*) loci (range = 6–8). Genotyping error rates per multilocus genotype (for samples in the final datasets) were higher for contemporary samples (allelic dropout = 17.0%; false alleles = 3.5%) than historical samples (allelic dropout = 7.9%; false alleles = 1.6%), but both were relatively low. Consequently, the probability of observing a genotyping error in the consensus genotype (i.e., [allelic dropout rate +false allele rate]^replicates^) was low at the average number of replicates performed for contemporary (5.25 ± 0.08 *SE*) and historical (7.69 ± 0.27 *SE*) individuals. We did not detect evidence of contamination in any extraction or PCR negatives.

### 3.2 Genetic diversity and population genetic structure

Only 4% and 2% of pairwise comparisons among contemporary and historical kit foxes, respectively, had a coefficient of relatedness>0.45. We removed 31 contemporary and seven historical individuals to minimize the influence of closely related individuals on subsequent genetic diversity and population genetic structure analyses. Resulting sample sizes for analyses of genetic diversity and population genetic structure (i.e., 45 contemporary and 42 historical foxes) exceeded the number of individuals required to accurately characterize population‐level genetic diversity (Hale et al., 2012).

We observed a similar number of alleles for historical and contemporary populations across loci (Table 1). We found no evidence of differences in allele frequencies for historical samples when they were split temporally (Table 2), suggesting our historical samples represented a single genetic population. We did not detect evidence of departures from HWE for historical samples (Table 1). For contemporary samples, we detected departure from HWE at one locus (Table 1). We found no evidence of linkage disequilibrium across loci for either historical or contemporary populations.

**Table 1:** The number of alleles (*N*_A_), allelic richness (*A*_r_), observed (*H*_O_), and unbiased expected heterozygosity (*H*_E_), fixation index (*F*_IS_), and *P*‐value for the test of Hardy–Weinberg equilibrium (HWE) for nine microsatellite loci amplified for historical (Hist.) and contemporary (Cont.) kit foxes (*Vulpes macrotis*) sampled in western Utah from 1951 to 1969 and 2013 to 2014, respectively. Bold indicates a locus not in Hardy–Weinberg equilibrium at α = 0.05 following Bonferroni's corrections

Locus	*N*_A_		*A*_r_		*H*_O_		*H*_E_		*F*_IS_		HWE	
	Hist.	Cont.	Hist.	Cont.	Hist.	Cont.	Hist.	Cont.	Hist.	Cont.	Hist.	Cont.
CXX103	5	4	4.6	4.0	0.50	0.73	0.52	0.63	0.035	−0.179	0.091	0.124
FH2010	4	5	4.0	5.0	0.73	0.79	0.70	0.76	−0.048	−0.053	0.881	0.946
CPH3	3	3	2.7	3.0	0.40	0.35	0.41	0.44	−0.002	0.206	1.000	**0.004**
CXX250	5	6	5.0	5.3	0.39	0.65	0.45	0.57	0.105	−0.165	0.083	0.722
CXX377	11	12	10.3	10.3	0.81	0.70	0.85	0.81	0.033	0.120	0.279	0.021
FH2001	6	9	5.3	7.5	0.55	0.69	0.51	0.65	−0.094	−0.064	0.556	0.118
FH2054	6	5	5.9	4.8	0.69	0.58	0.71	0.68	0.015	0.140	0.894	0.026
FH2088	8	8	7.6	7.8	0.78	0.73	0.70	0.72	−0.128	−0.034	0.919	0.975
VVE‐M19	9	7	8.0	6.6	0.75	0.86	0.75	0.78	−0.025	−0.120	0.445	0.493
Mean	6.3	6.6	5.9	6.0	0.62	0.68	0.62	0.67	−0.012	−0.017		
*SE*	0.85	0.93	0.74	0.71	0.05	0.05	0.05	0.04	0.024	0.046		

**Table 2:** Results for *G* tests implemented in genepop for differences in allele frequencies (among nine microsatellite loci) between two (1951–1959 vs. 1961–1969) or three (1951–1955 vs. 1958–1962 vs. 1964–1969) temporal groups of historical kit foxes (*Vulpes macrotis*) from specimens sampled in western Utah from 1951 to 1969, and between all historical (1951–1969) and contemporary (2013–2014) kit foxes. Bold indicates a locus with significant genic differentiation between historical and contemporary populations at α = 0.05 following Bonferroni's corrections

Locus	Historical: 2 Groups		Historical: 3 Groups		Historical versus Contemporary	
	*p*‐Value	*SE*	*p*‐Value	*SE*	*p*‐Value	*SE*
CXX103	0.332	0.006	0.860	0.004	0.051	0.003
FH2010	0.272	0.006	0.796	0.005	0.041	0.003
CPH3	0.146	0.005	0.703	0.006	0.281	0.005
CXX250	0.023	0.002	0.143	0.005	0.482	0.007
CXX377	0.259	0.008	0.167	0.007	0.021	0.002
FH2001	0.798	0.004	0.366	0.009	0.069	0.004
FH2054	0.083	0.004	0.359	0.008	**0.005**	**0.002**
FH2088	0.895	0.003	0.599	0.008	0.105	0.006
VVE‐M19	1.000	0.000	0.749	0.008	0.426	0.012

**Figure 1:**
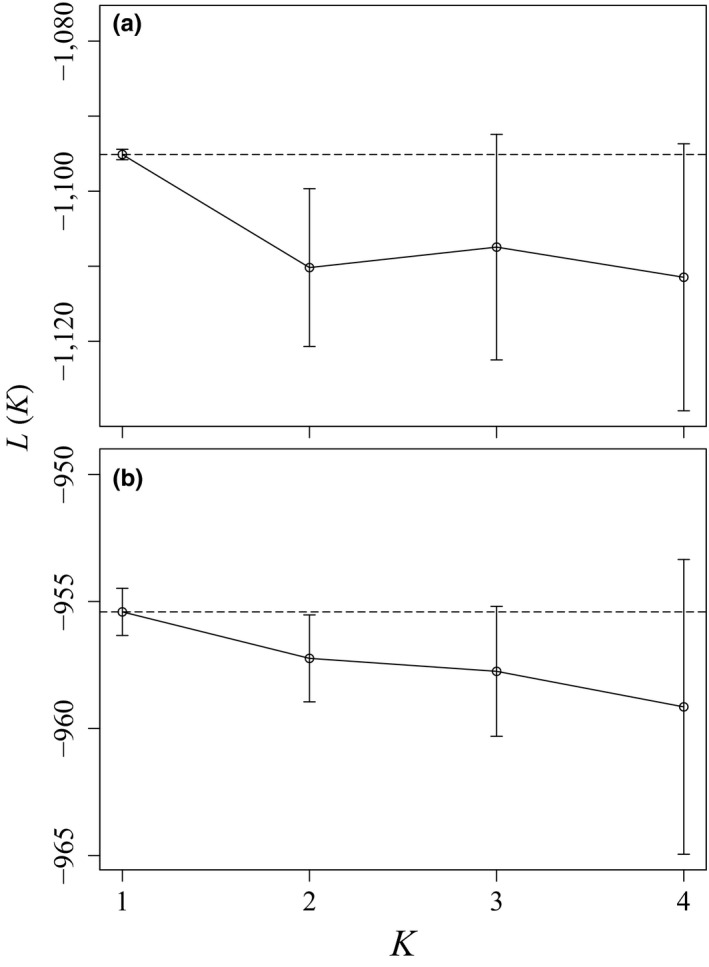
The most likely number of genetically distinct clusters (*K*) of kit foxes (*Vulpes macrotis*) during (a) contemporary (2013–2014) and (b) historical (1951–1969) sampling in western Utah based on the program structure. The mean maximum likelihood [L(*K*)] supported *K = *1 in both contemporary and historical populations; ancestry plots (not shown) support these conclusions, with individuals having ancestry split evenly among populations when *K* > 1. The horizontal dashed line represents the highest mean L(*K*) observed and vertical bars on L(*K*) are ±1 *SD*.

The structure analysis of contemporary samples suggested that our sample represented a single genetic population, with the mean maximal value of L(*K*) at *K* = 1 (Figure 1). Similarly, we found no evidence of genetic structure within our historical kit fox specimens (Figure 1). Ancestry values from structure confirmed these results; individual ancestries were split approximately evenly among populations when >1 population was considered.

We found no evidence of significant differences in *A*_r_ (*t* = −0.28, *df* = 8, *p* = 0.79) or *H*_E_ (*t* = −2.29, *df* = 8, *p* = 0.051) between historical and contemporary populations. For both historical and contemporary populations, mean *H*_O_ was comparable to mean *H*_E_, and mean *F*_IS_ was not different from zero, aligning with tests for HWE (Table 1).

### 3.3 Effective population size and identification of immigrants

**Figure 2:**
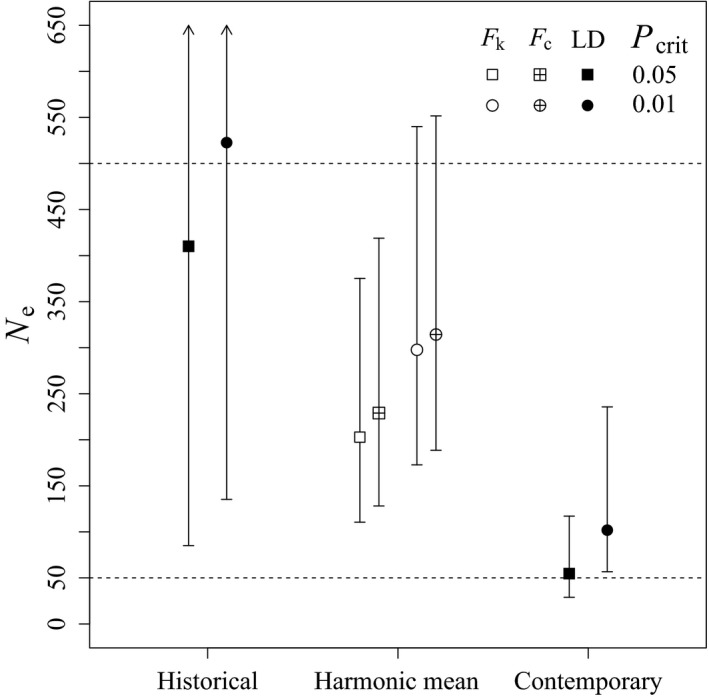
Estimates of effective populations size (*N*_e_) and harmonic mean *N*_e_ based on single‐sample linkage disequilibrium (LD) methods and two‐sample temporal methods (*F*_k_ and *F*_c_), respectively, for historical (1951–1969) and contemporary (2013–2014) kit foxes (*Vulpes macrotis*) sampled in western Utah. Rare alleles occurring at frequencies below the critical values (*P*_crit_) were removed. Confidence intervals (95%) are based on the jackknife‐across samples method with arrows indicating that the upper bound it was indistinguishable from infinite. Horizontal dashed lines highlight the levels of the 50/500 rule for reducing the risk of inbreeding depression and maintaining adaptive potential

Linkage disequilibrium estimates of historical *N*_e_ were 5.1–7.5 times higher than estimates of contemporary *N*_e_ (Figure 2). Upper confidence bounds of historical linkage disequilibrium *N*_e_ estimates were indistinguishable from infinity. Considering the harmonic mean of estimates across critical values, the estimate of historical *N*_e_ was 460 (95% CI = 104.5–∞). As expected, both temporal methods produced similar estimates of *N*_e_ that were intermediate with respect to the linkage disequilibrium estimates (Figure 2). Temporal estimates of *N*_e_ with a critical value = 0.01 were slightly higher than those with a critical value = 0.05, and upper confidence bounds exceeded 500 (Figure 2). Still when the harmonic means and confidence intervals were calculated across critical values for estimates of *N*_e_ from temporal methods, neither method produced estimates with an upper bound exceeding 500 (*F*_k_: *N*_e_ = 241, 95% CI = 134.8–442.8; *F*_c_: *N*_e_ = 265, 95% CI = 152.6–476.2). Linkage disequilibrium estimates of contemporary *N*_e_ were well below the *N*_e_ = 500 threshold and near the critical threshold of 50 individuals (Figure 2). The harmonic mean of contemporary linkage disequilibrium estimates of *N*_e_ across critical values was 71 (95% CI = 38.4–156.5).

Among 76 contemporary individuals, two individuals (M:F ratio = 1:1) were identified as having a probability of population membership <1%, whereas five individuals (M:F ratio = 4:1) had a probability of population membership <5%. Thus, naïve estimates of contemporary immigration ranged from 2.6% to 6.6%. Among 49 historical kit foxes, four individuals had a probability of population membership <5% (i.e., naïve estimate of historical immigration = 8.2%).

## 4 DISCUSSION

Comparing historical specimens from NHCs with contemporary populations can provide insights into how natural and anthropogenic changes influence populations and inform conservation (Johnson, Bellinger, Toepfer, & Dunn, 2004; Miller & Waits, 2003; Rosenbaum, et  al., 2000). For conservation, historical specimens would ideally characterize the population prior to significant human impacts (Hare et al., 2011). Kit fox densities in western Utah were relatively stable during the 1950s and 1960s (10–22 foxes/100 km^2^; Egoscue, 1956, 1962, 1975 ) and were 5–11 times higher than contemporary densities (two foxes/100 km^2^; Lonsinger et al., 2018). We used historical specimens that aligned temporally with the period preceding a precipitous decline in kit fox abundance to evaluate changes in genetic parameters following landscape and community dynamic changes. We found evidence that *N*_e_ of kit foxes decreased substantially, and that contemporary *N*_e_ was precariously close to levels identified as being at risk of inbreeding depression and local extinction. Interestingly, we found no significant decrease in genetic diversity associated with declining abundance and *N*_e_, and hypothesized that genetic diversity was maintained by undescribed immigration from other populations.

Franklin's (1980) 50/500 rule proposed thresholds for *N*_e_ required to avoid inbreeding depression in the short term (~5 generations; *N*_e_ ≥ 50) and to ensure long‐term adaptive potential and persistence (in perpetuity; *N*_e_ ≥ 500). Genetic evidence from wild populations has suggested these thresholds are too low, and Frankham, Bradshaw, and Brook (2014) suggested the 50/500 rule be increased to 100/1,000. Single‐sample estimates of contemporary inbreeding *N*_e_ indicated the kit fox population under study was well below the long‐term thresholds and at or below short‐term thresholds. Estimates of *N*_e_ from large populations are expected to be less precise than those from smaller populations (Palstra & Ruzzante, 2008). Upper confidence limits of our historical inbreeding *N*_e_ estimates were infinite. Still, point estimates and lower confidence limits suggested that historical inbreeding *N*_e_ was similar to levels required to maintain adaptive potential under the 50/500 rule, but not the 100/1,000 rule. As we expected, two‐sample (temporal) estimates of variance *N*_e_ were intermediate and suggested the harmonic mean *N*_e_ between sampling periods was likely greater than the lower thresholds to avoid inbreeding depression, but less than thresholds to maintain adaptive potential. Collectively, these findings suggest *N*_e_ of the kit fox population has decreased from relatively secure levels prior to 1970 to levels that warrant increased conservation attention.

As *N*_e_ decreases, inbreeding and drift are both expected to lead to declines in genetic diversity (Frankham, 2005). For example, genetic diversity declined significantly in association with declining *N*_e_ in mountain lions (*Puma concolor*; Holbrook, Deyoung, Tewes, & Young, 2012) and greater prairie chickens (*Tympanuchus cupido*; Johnson et al., 2004). Similarly, grizzly bears in Yellowstone experienced significant declines in genetic diversity with more gradual declines in *N*_e_ (Miller & Waits, 2003), and critically endangered arctic foxes (*Alopex lagopus*) experienced significant decreases in genetic diversity during a population bottleneck (Nyström, Angerbjo, & Dalen, 2006). Comparing the harmonic means of the linkage disequilibrium inbreeding *N*_e_ estimates for our historical to contemporary kit fox populations suggested an 85% decline. Despite observed declines in *N*_e_, we found no evidence of declining genetic diversity. Genetic diversity in populations with small *N*_e_ may be maintained by immigration (Palstra & Ruzzante, 2008), and even low levels of gene flow (e.g., one migrant per generation) can result in the maintenance of local genetic diversity at levels comparable to the entire metapopulation (Hare et al., 2011). Similar patterns of declining *N*_e_ with stable genetic diversity have been observed in Atlantic salmon (*Salmo salar*) and attributed to immigration from neighboring metapopulations (Consuegra, Verspoor, Knox, & García De Leániz, 2005; Fraser, Jones, McParland, & Hutchings, 2007). We initially suspected our population was isolated due to the topography and landcover surrounding the study extent. The nearest sites outside of our study extent with recent evidence of kit fox occurrences were ≥25 km away (Richards, 2017), and no telemetered kit foxes being monitored by another study were documented to have dispersed beyond our study extent (B. Kluever, personal communication). While our population genetic structure analysis did not reveal population subdivisions, we found evidence of immigrants in the population and estimates of contemporary immigration were not substantially lower than estimates of historical immigration. Although this does not alleviate the concerns associated with low observed *N*_e_, it does suggest that the issues associated with the lower risk thresholds for inbreeding depression may be partially mitigated by intermittent or low levels of gene flow and highlights the importance of identifying corridors for conservation. With the low estimated *N*_e_ of the population, genetic stochasticity is likely to become important if the population becomes isolated (Palstra & Ruzzante, 2008).

Both single‐sample linkage disequilibrium and temporal methods make simplifying assumptions that may influence estimates. Temporal methods assume discrete generations, yet many studies (including ours) apply these methods to species with overlapping generations (Waples & Yokota, 2007). Bias associated with overlapping generations is reduced when there is ≥5 generations between temporal samples, and negligible when ≥10 generations separate samples (Waples & Yokota, 2007). Our samples were separated by ~13 generations, and we do not expect significant bias associated with overlapping generations. When population size changes, temporal methods estimate the harmonic mean *N*_e_ over time between samples. Linkage disequilibrium methods assume constant population size (Waples & Yokota, 2007). Kit fox abundance certainly changed between historical and contemporary populations, and we interpreted variance *N*_e_ as the harmonic mean *N*_e_ between sampling periods. Population size was relatively stable during each sampling period used for linkage disequilibrium *N*_e_ estimates (Egoscue, 1962, 1975 ; Lonsinger et al., 2018). Gene flow may also influence estimates of *N*_e_. When migration rate is low, temporal methods produce estimates with minimal bias (Nei & Tajima, 1981) and linkage disequilibrium methods provide robust estimates of *N*_e_ (Waples & Do, 2010). We detected only low levels of immigration and therefore our estimates of *N*_e_ should represent the *N*_e_ of the population under study, rather than the entire metapopulation (Nei & Tajima, 1981). Finally, small sample sizes can lead large biases in *N*_e_ estimates, but samples of 50–100 are sufficient to produce unbiased estimates (Waples & Yokota, 2007). Our sample sizes were comparable to this range, and we did not expect any significant biases associated with sample size.

Genetic comparisons between historical and contemporary populations can be used to assess impacts of management actions, evaluate size of populations during bottlenecks, infer population risk, and inform management and conservation (Holbrook et al., 2012; Johnson et al., 2004; Miller & Waits, 2003; Nyström et al., 2006). In the absence of long‐term studies, NHCs facilitate these comparisons. Despite their importance, support for NHCs has been declining (Dalton, 2003). Our study highlights the importance of NHCs to conservation and demonstrates how a historical baseline can alter conclusions from those based exclusively on contemporary data. Such comparisons have been limited, likely due to the lack of historical specimens that align spatially and temporally with the research objectives (Wandeler, Hoeck, & Keller, 2007). Our study was facilitated by historical research on kit foxes at Dugway (Egoscue, 1956, 1962, 1975 ). There is often concern with historical specimens regarding the reliability of their spatial data (Wandeler et al., 2007). The spatial resolution of our historical specimens was limited to the county of collection, but we had relatively high confidence in these locations and our population genetic structure analyses confirmed that specimens were all from the same population. Our historical specimens aligned with the period before kit fox populations declined and therefore should adequately represent historical genetic diversity.

Much of our broad understanding of kit fox ecology comes from the early research at Dugway (Egoscue, 1956, 1962 ). Presumably, kit foxes have been declining at the site for ~40 years. Over the past two decades, considerable research effort has been invested into understanding the influence of changing landscape and community dynamics on kit foxes. For example, these studies have investigated the responses of kit foxes to changing vegetation (Arjo et al., 2007), water availability (Kluever & Gese, 2017), prey communities (Byerly et al., 2018), and intraguild predator abundances (Lonsinger et al., 2017). Despite this long research record and strong efforts on the parts of managers and researchers, few practical conservation actions have been identified for kit foxes. One critical aspect of kit fox conservation that has not previously been investigated is the genetic health of the population. Our study began to address this important topic, advanced our understanding of kit fox ecology, and provided insights that can inform conservation. Our results confirm that the precipitous decline in kit fox abundance has resulted in a sharp decline in *N*_e_ to levels predicted to put the population at imminent risk (within ~5 generations, or ~17 years) of inbreeding depression. In contrast to these findings, we found no evidence of declining genetic diversity when considering the historical baseline data for genetic diversity. We hypothesize that genetic diversity has been maintained through low or intermittent immigration and argue that conservation efforts should prioritize assessing connectivity further. Identifying, maintaining, and potentially promoting or restoring gene flow with adjacent populations would likely decrease risks to kit foxes from short‐term genetic stochasticity and help promote long‐term conservation. Advancements in the field of landscape genetics provide a framework for effectively evaluating patterns of gene flow and identifying key corridors for conservation (Balkenhol, Cushman, Storfer, & Waits, 2016).

## CONFLICT OF INTEREST

None declared.

## AUTHOR CONTRIBUTIONS

RCL and LPW conceived the research. RCL and JRA performed data collection and conducted laboratory procedures. All authors conducted analyses. RCL led the manuscript preparation. JRA and LPW assisted with manuscript preparation and review.

## DATA ACCESSIBILITY

Genetic data are available from the Dryad Digital Repository: https://doi:10.5061/dryad.kc5j299.
